# Text Neck Joint Position Error Among Taibah University Students, Saudi Arabia—Cross-Sectional Design

**DOI:** 10.3390/healthcare14101320

**Published:** 2026-05-12

**Authors:** Abdulrhman Mashabi, Walaa M. Ragab, Shahad B. Aljohani, Rand A. Aljohani, Lama A. Almadani, Fai A. Alsharif, Jumanah A. Aburibiyyah, Marwan M. A. Aljohani, Abdullah Al-Shenqiti

**Affiliations:** Department of Physical Therapy, College of Medical Rehabilitation Sciences, Taibah University, Madinah 42353, Saudi Arabia

**Keywords:** joint position error, cervical proprioception, text neck, forward head posture, laser beam, inclinometer, smartphone goniometer

## Abstract

**Background:** Proprioceptive input from cervical muscles plays a vital role in postural control and coordinated movement. A defect in cervical proprioception, known as joint position error (JPE), is often associated with neck pain. However, the presence of JPE in asymptomatic individuals with varying severities of text neck or forward head posture (FHP) remains underexplored. This study aimed to investigate the presence and correlation of JPE in healthy female university students with different levels of text neck severity. **Methods:** A cross-sectional study was conducted on 68 female students aged 18–25 years. Participants were categorized into four groups (normal posture, mild, moderate, and severe text neck) through visual observational assessment. JPE was measured in sitting and standing positions using three tools: an inclinometer, a smartphone-based goniometer, and a laser beam. Correlational and comparative analyses were conducted across all groups and measurement tools. **Results:** The results demonstrated the presence of JPE in all groups, regardless of text neck severity, with no statistically significant differences between them. Additionally, correlation analysis showed no or weak non-significant relationships between JPE and text neck severity across all measurement tools. **Conclusions:** Cervical JPE may be present in young adults regardless of their text neck posture, and no significant correlation was found between the severity of text neck and proprioceptive deficit. These findings suggest that text neck alone may not be a predictive factor for impaired cervical proprioception in asymptomatic individuals. Early screening remains important, but further research is needed to understand contributing factors and long-term implications.

## 1. Introduction

Proprioception refers to the continuous somatosensory input provided to the central nervous system (CNS) by peripheral receptors located in muscles, joint capsules, ligaments, and articular structures. This sensory input plays a crucial role in regulating posture, spatial orientation, and balance by enabling the body to coordinate movements and maintain joint stability [1]. Efficient neuromuscular control and balance against gravitational forces depend heavily on intact proprioceptive feedback. In the cervical spine, proprioception is modulated by cervical mechanoreceptors, visual-ocular input, and the vestibular system. Disruption of any of these systems may result in proprioceptive deficits, which are commonly implicated in various neck pathologies [2]. One of the most frequently reported causes of impaired cervical proprioception is neck pain. Altered afferent signals originating from painful cervical structures may lead to disturbed proprioceptive feedback, contributing to sensorimotor dysfunction. This condition is particularly prevalent in older adults, where age-related degenerative changes often result in chronic neck pain and associated proprioceptive deficits [3]. Furthermore, whiplash-associated disorders have been recognized as a significant contributor to cervical joint position sense errors, with evidence demonstrating persistent impairments in proprioceptive acuity following such trauma [4]. In recent years, attention has shifted toward non-traumatic causes of proprioceptive dysfunction, particularly forward head posture (FHP), commonly referred to as “text neck” [5]. Text neck is a type of forward head posture characterized by hyperextension of the upper cervical spine (C1–C3) and flexion of the lower cervical spine (C4–C7). This condition may lead to altered muscle activation, impaired balance, respiratory dysfunction, and sleep disturbances. Its prevalence among university students in Saudi Arabia has been reported to reach 68.1%, with a strong association with prolonged electronic device use and sedentary behavior [6]. The assessment of cervical proprioception is typically conducted using joint position error (JPE), which reflects the accuracy with which an individual can return the head to a neutral position following cervical movement. Tools used to measure JPE include both subjective and objective methods [7]. While observational approaches may provide preliminary insights, objective instruments such as laser devices, digital inclinometers, and smartphone-based goniometers offer more reliable and reproducible measurements of cervical joint repositioning accuracy [2,8,9]. Despite the growing body of evidence linking neck pain to proprioceptive dysfunction, research examining these deficits in asymptomatic individuals, particularly those with forward head posture, remains limited. Understanding the distribution and severity of proprioceptive deficits in individuals without neck pain but with postural deviations, such as text neck, has important clinical implications. Early detection and targeted interventions may help reduce the risk of future musculoskeletal disorders. Moreover, quantifying the relationship between JPE and the severity of FHP may inform preventive strategies and guide clinical management. To date, no studies have investigated cervical proprioceptive function in university students in Saudi Arabia across varying levels of text neck severity. Therefore, this study aimed to examine whether proprioceptive deficits differ among students with different degrees of text neck and to evaluate the relationship between cervical JPE and postural deviation in this population.

## 2. Methods

### 2.1. Participants and Study Procedures

This observational cross-sectional study was conducted at Taibah University between 31 January 2022 and 18 April 2022. The study protocol was reviewed and approved by the Institutional Review Board (IRB) of Medical Rehabilitation Sciences (Approval No: CMR-PT-2022-01). All procedures were carried out in accordance with the ethical standards of the institutional research committee and the principles of the 1964 Declaration of Helsinki and its subsequent amendments. Written informed consent was obtained from all participants prior to their inclusion in the study. A convenience sample of 68 unblinded healthy female university students aged 18–25 years was recruited. Participants were enrolled from the College of Medical Rehabilitation Sciences, the Faculty of Applied Medical Sciences, and the Faculty of Business Administration at Taibah University through posters, word-of-mouth invitations, and announcements via WhatsApp student groups. Participants were eligible if they were healthy female students without current neck pain and with no history of cervical or spinal surgery, whiplash injury, or congenital spinal deformities. Exclusion criteria included scoliosis, significant thoracic kyphosis, torticollis, balance disorders, use of hearing aids, rheumatologic conditions, persistent respiratory problems, or a history of cervical spine fractures or traumatic injuries. Following recruitment, participants were categorized into four groups based on the severity of text neck posture: normal (no text neck), mild, moderate, and severe. Classification was initially performed through visual postural assessment by an independent team of trained researchers and subsequently verified by the main research team to ensure consistency [10]. All assessments were conducted in a dedicated research room in the Physiotherapy Laboratory at the College of Medical Rehabilitation Sciences (female campus), Taibah University. Environmental noise and distractions were minimized. Data collection was carried out 3–4 days per week between 11:00 a.m. and 3:00 p.m. Each participant completed a single 15-min assessment session, which included measurements of cervical joint position error (JPE) using three instruments: a bubble inclinometer, a smartphone-based goniometer (Clinometer application on iPhone), and a laser beam device. Measurements were obtained in both sitting and standing positions using standardized procedures [11].

### 2.2. Assessment Tools

**Figure 1:**
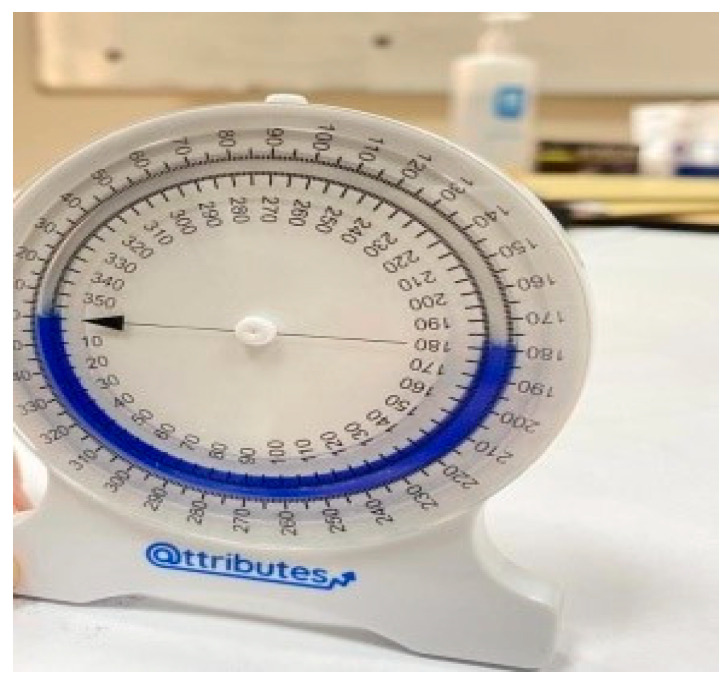
The inclinometer.

**Figure 2:**
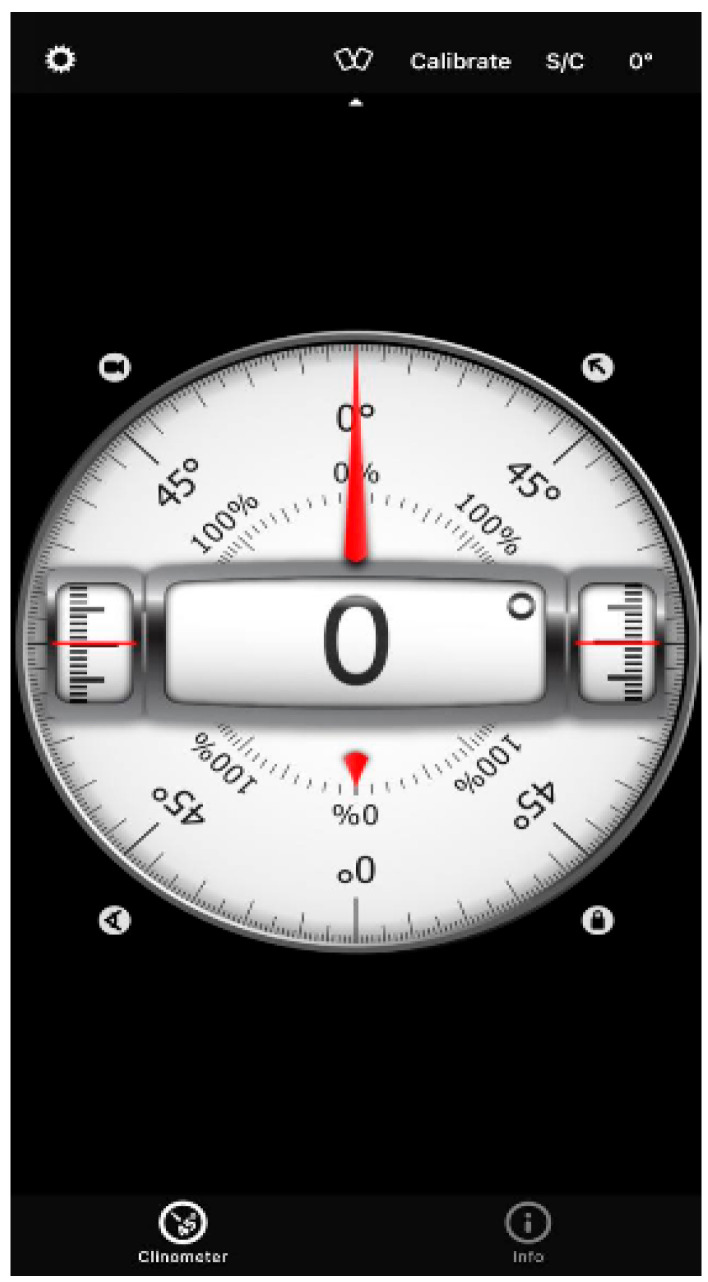
Smartphone application goniometer.

**Figure 3:**
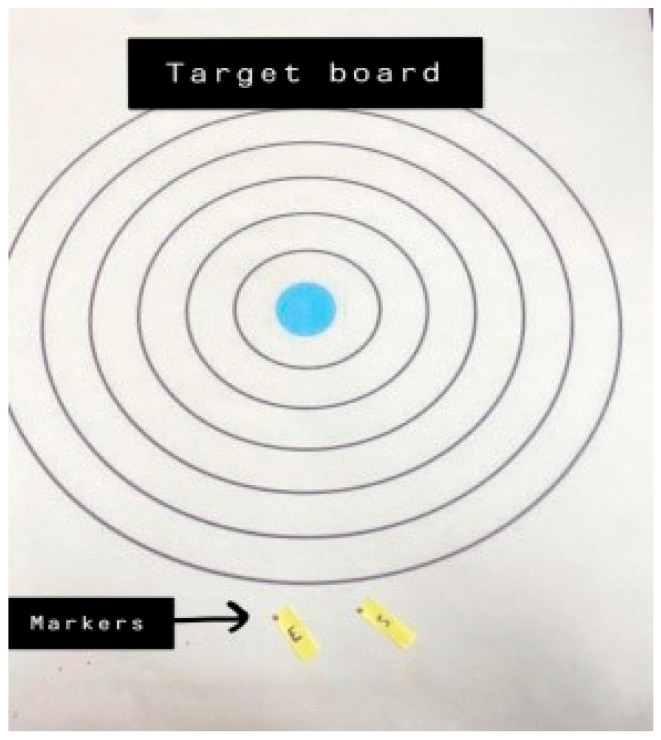
The target board.

**Figure 4:**
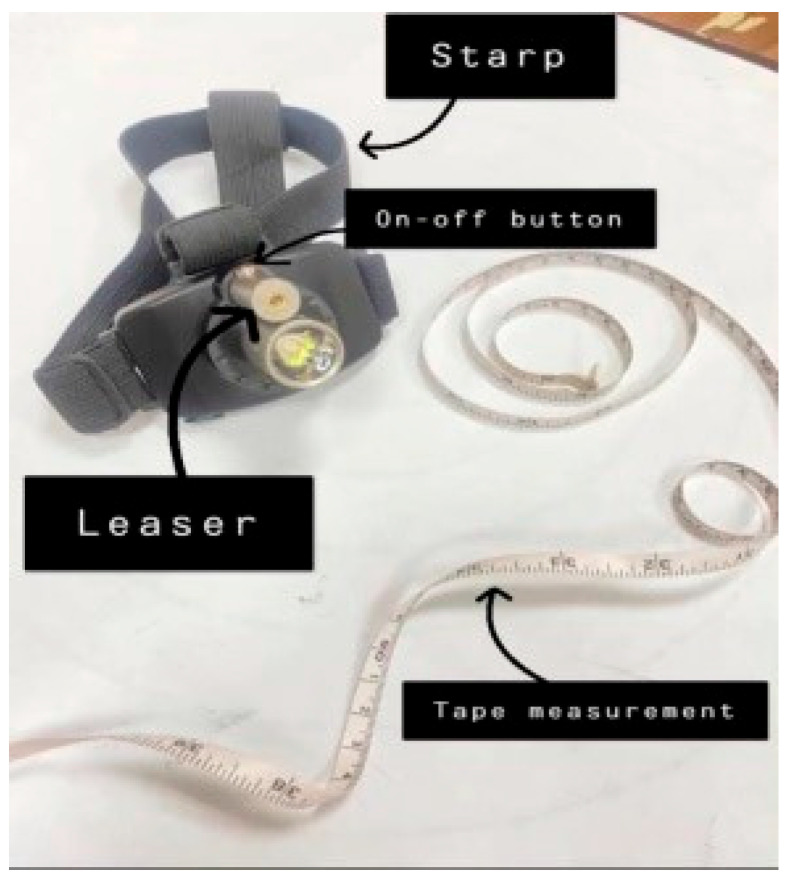
Components of the laser beam device.

**Figure 5:**
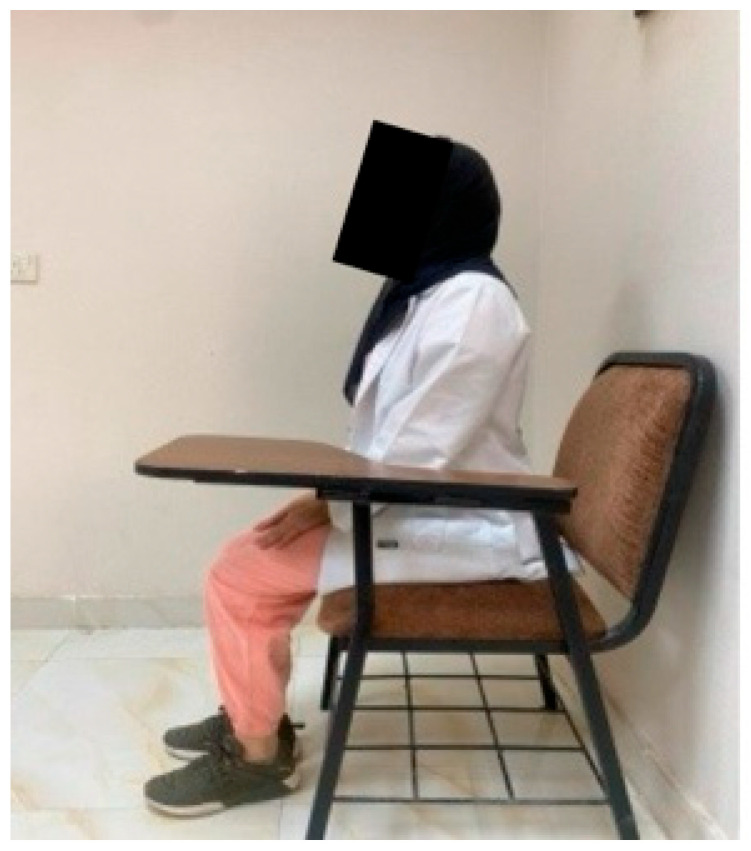
The ideal sitting position for JPE assessment.

**Figure 6:**
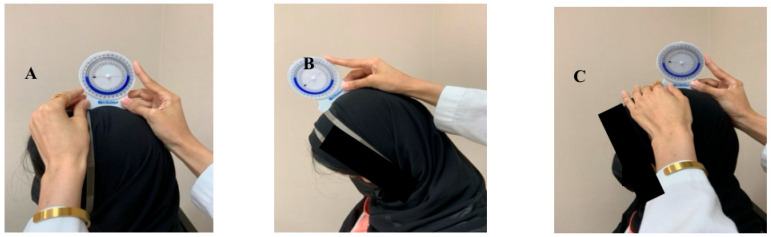
Inclinometer joint position error for neck flexion. (**A**) starting position, (**B**) full range of neck flexion, (**C**) restart neck position.

**Figure 7:**
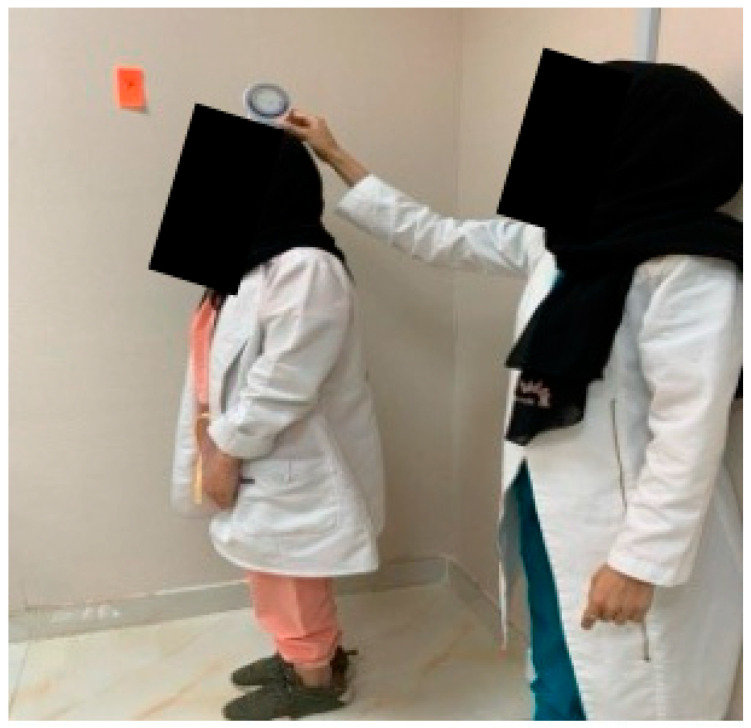
Standing position for JPE.

**Figure 8:**
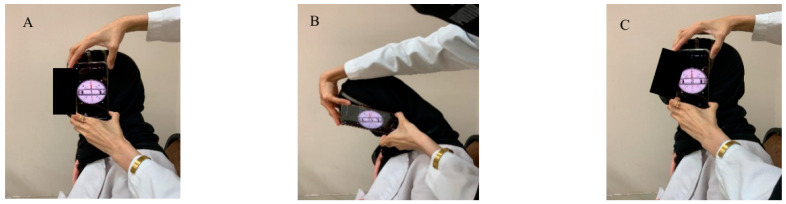
Smartphone goniometer joint position error for neck flexion (**A**) starting position, (**B**) full neck flexion, (**C**) restart neck position.

**Figure 9:**
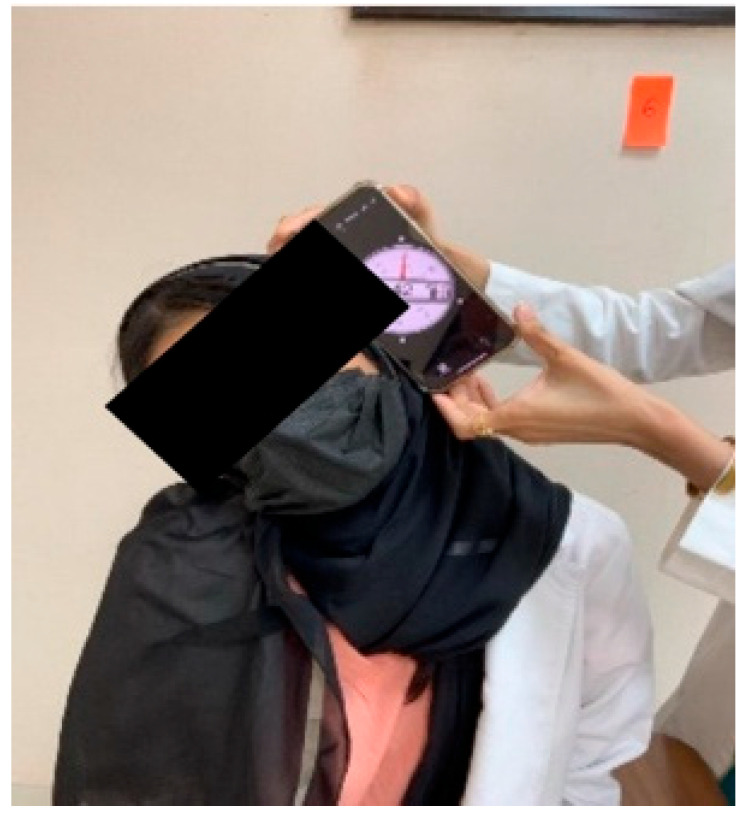
Smartphone side bending JPE.

**Figure 10:**
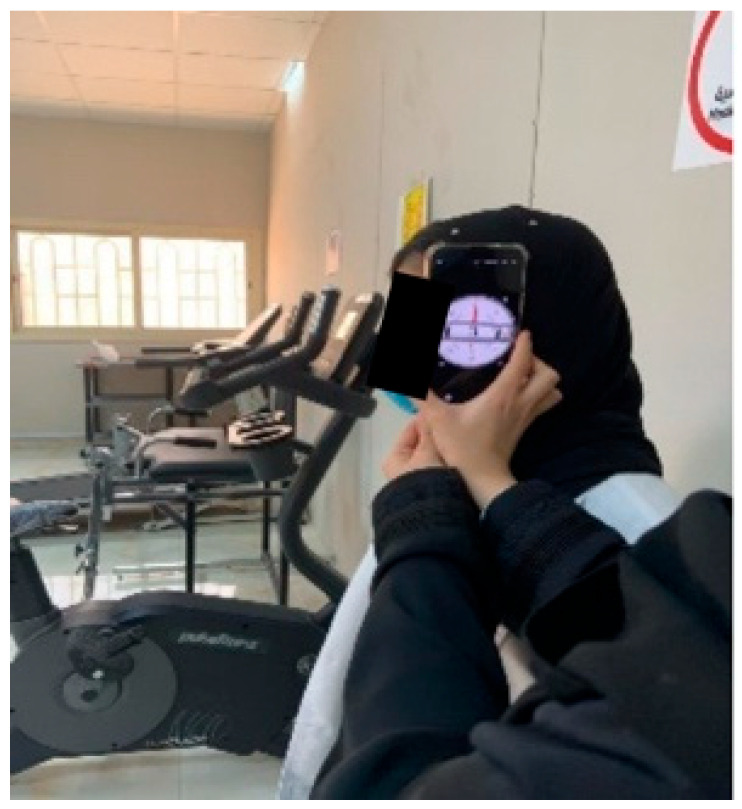
Standing position JPE.

**Figure 11:**
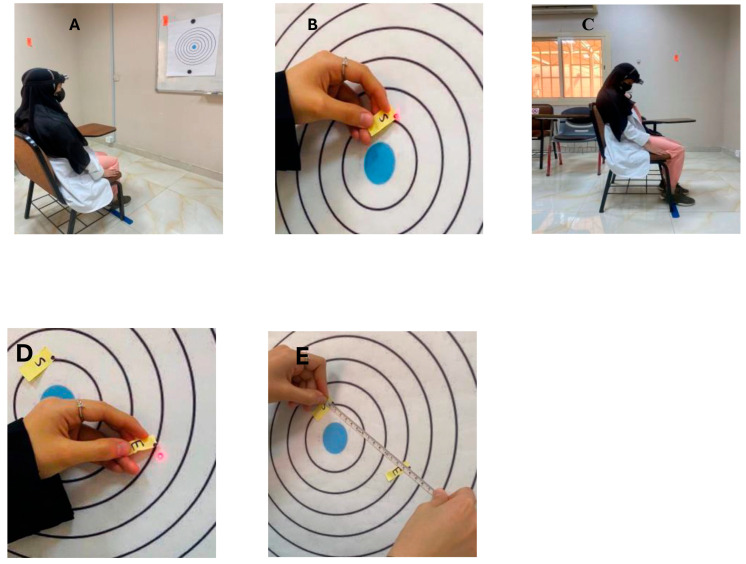
Laser beam joint position error for neck flexion (**A**) starting sitting position, (**B**) The start position (point S), (**C**) full range of neck flexion with closed eyes, (**D**) restart position (point E), (**E**) Measure the distance between the start and end point).

**Figure 12:**
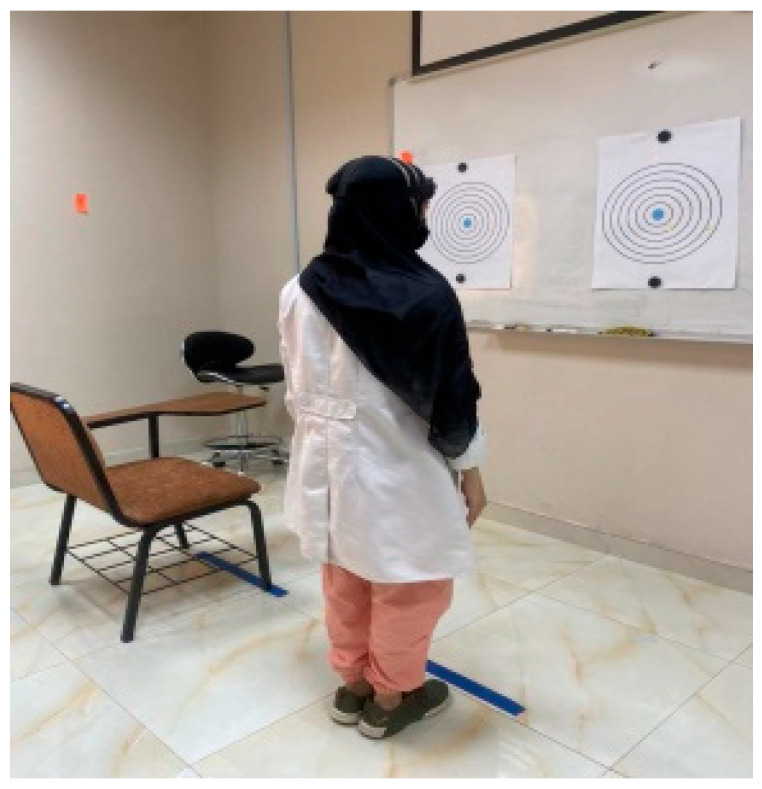
Standing position for JPE using laser beam.

A bubble inclinometer (Model 12-1056, Fabrication Enterprises Inc., White Plains, NY, USA) was used to measure cervical angles relative to gravity. This non-invasive tool is widely used for assessing cervical proprioception and has demonstrated strong reliability and validity [8,11] (Figure 1). The smartphone goniometer consisted of an iPhone equipped with the Clinometer application (version 4.9.4), which utilizes an internal three-axis accelerometer to measure cervical angles in the sagittal and frontal planes. This application has demonstrated moderate to good validity and intra-rater reliability for assessing cervical range of motion [12] (Figure 2). A laser beam device (Lalomo rechargeable LED headlamp, Lalomo, Jiaxing, China) was used to assess joint position error (JPE). The laser was mounted on a head strap worn on the forehead, and a target board was positioned at a fixed distance of 1 m in front of the participant to record the deviation between the initial and repositioned head positions (Figure 3 and Figure 4). This method has been supported as a reliable approach for proprioception assessment [6,13]. Text neck severity was classified using observational analysis based on a vertical plumb line to assess the alignment between the tragus of the ear and the acromioclavicular (AC) joint. Normal posture was defined when the tragus was vertically aligned with the AC joint. Mild text neck was identified when the tragus was positioned anterior to the AC joint, while the posterior ear remained relatively aligned. Moderate text neck was defined when both the tragus and posterior ear aligned near the shoulder edge, whereas severe text neck was classified when both landmarks were positioned anterior to the shoulder line [10]. For JPE assessment, each participant was positioned in an upright sitting posture with hips and knees flexed at 90°, feet flat on the floor, and the spine in a neutral position. Participants were instructed to relax their neck muscles, memorize the starting head position, and reproduce it following the movement. All measurements were performed with the eyes closed to eliminate visual input. JPE was assessed in four cervical movement directions: flexion, extension, right lateral bending, and left lateral bending. Measurements were conducted using the inclinometer, smartphone goniometer, and laser beam device in both sitting and standing positions under standardized conditions. For each movement direction and each testing condition, one trial was performed and recorded, with a brief pause allowed between movements to minimize fatigue. For inclinometer and smartphone goniometer assessments, the device was positioned at the top of the head or beside the ear, respectively. Participants performed a full active cervical movement and then attempted to return to the initial position. The starting angle (“Start”) and the reproduced angle (“Restart”) were recorded. JPE was calculated as the absolute difference between these two values (in degrees). This procedure was applied in both sitting and standing positions [8,12] (Figure 5, Figure 6, Figure 7, Figure 8, Figure 9 and Figure 10). For the laser beam device, a head-mounted laser was secured on the participant’s forehead, and the participant was positioned 1 m from a wall-mounted target board. After performing the required cervical movement with eyes closed, the participant attempted to return to the initial position. The deviation between the initial laser point (point S) and the final repositioned point (point E) was measured using a metric tape. JPE was calculated as the linear displacement between these two points (in centimeters). This procedure was conducted in both sitting and standing positions [14] (Figure 11 and Figure 12).

### 2.3. Sample Size

**Figure 13:**
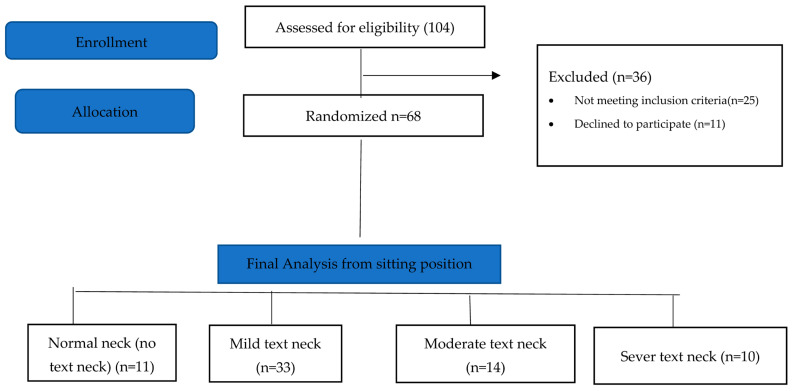
The flow chart from the sitting position assessment.

Sample size calculation was performed using G*Power software (version 3.1) based on a pilot study involving 16 participants (4 per group). The primary outcome was flexion JPE measured using the laser beam device in the standing position. Based on an effect size of 0.85, an alpha level of 0.05, a statistical power of 80%, and degrees of freedom of 3, the required sample size was estimated to be 46 participants (approximately 12 per group). The final sample size of 68 participants exceeded this requirement. The participant flow diagram is presented in Figure 13.

### 2.4. Data Analysis

Data were entered into Microsoft Excel 2019 and analyzed using XLSTAT 2022. Statistical significance was set at *p* < 0.05. The normality of data distribution and homogeneity of variance were assessed using the Shapiro–Wilk and Levene tests, respectively. Due to non-normal data distribution and unequal group sizes, the Kruskal–Wallis test was used to compare JPE across the four groups. Spearman’s rank correlation was used to assess the association between text neck severity and JPE across all cervical movement directions and measurement tools [15].

## 3. Results

**Figure 14:**
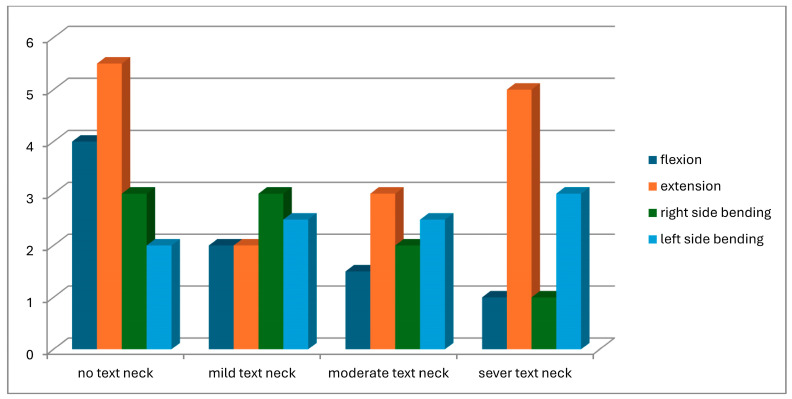
Neck movements Inclinometer median values comparison for JPE from the standing position.

**Figure 15:**
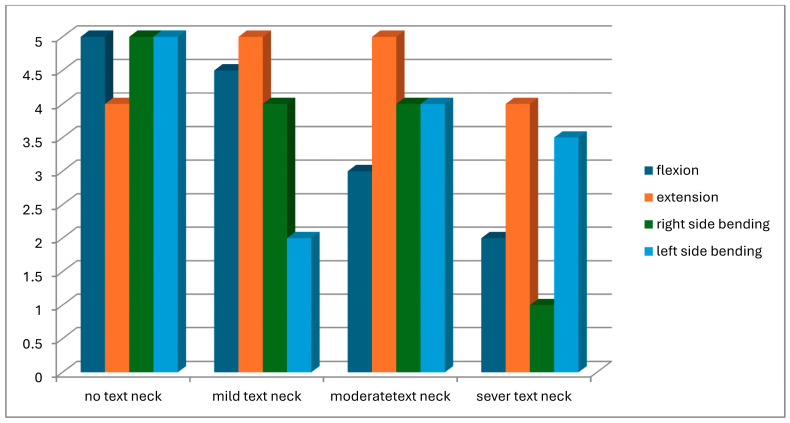
Neck movements smart phone goniometer median values comparison for JPE from the standing position.

**Figure 16:**
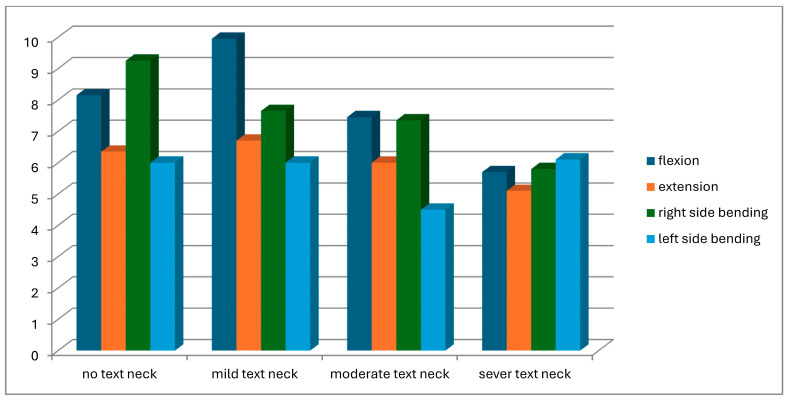
Neck movements laser beams median values comparison for JPE from the standing position.

**Figure 17:**
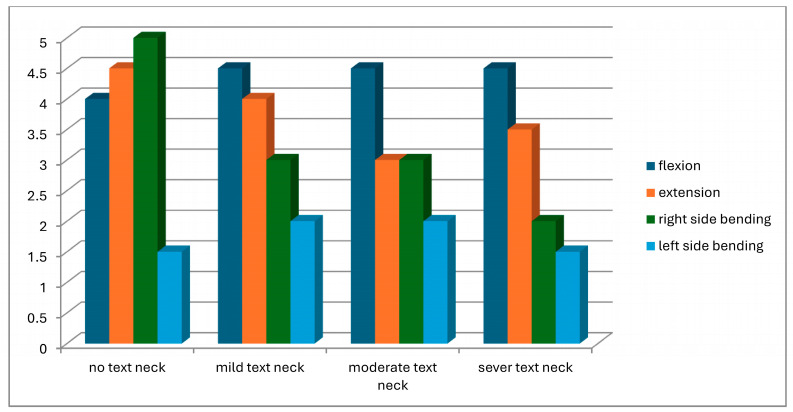
Neck movements Inclinometer median values comparison for JPE from the sitting position.

**Figure 18:**
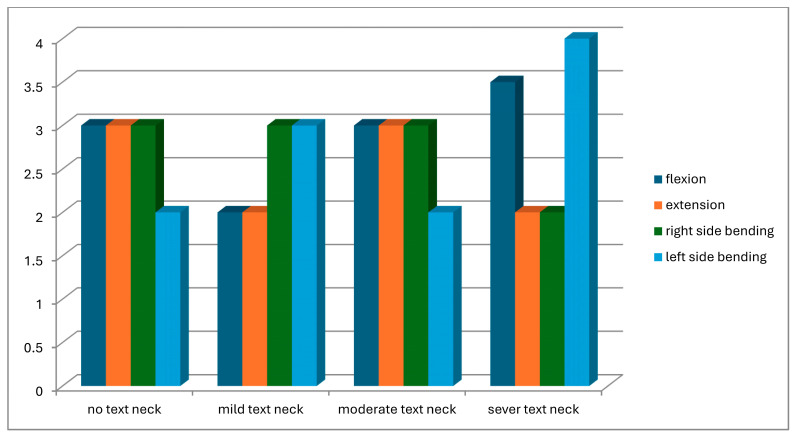
Neck movements smart phone goniometer median values comparison for JPE from sitting position.

**Figure 19:**
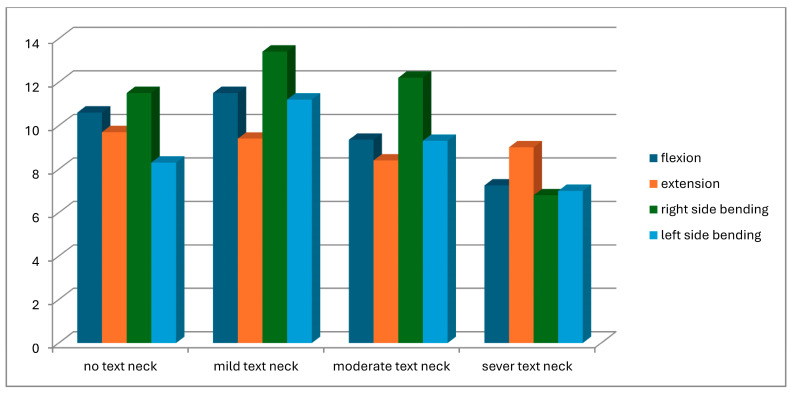
Neck movements laser beam median values comparison for JPE from the sitting position.

A total of 104 students were screened for eligibility. Of these, 25 did not meet the inclusion criteria, and 11 withdrew due to scheduling conflicts with lecture times. Consequently, 68 participants completed the study and were assessed for text neck posture in both standing and sitting positions. Based on observational assessment in the standing position, participants were classified into four groups: normal posture (n = 8), mild text neck (n = 37), moderate text neck (n = 14), and severe text neck (n = 9). Classification based on the sitting position is illustrated in Figure 13. The demographic characteristics of the four groups, classified according to standing posture, are presented in Table 1. Statistical analysis using the Kruskal–Wallis test revealed no statistically significant differences among groups in age (*p* = 0.22), weight (*p* = 0.11), height (*p* = 0.93), or body mass index (BMI) (*p* = 0.05), indicating baseline comparability. Similarly, demographic data based on sitting posture classification (Table 2) showed no significant differences among groups in age (*p* = 0.26), weight (*p* = 0.11), height (*p* = 0.48), or BMI (*p* = 0.06). These findings confirm demographic homogeneity across all groups. Joint position error (JPE) in the standing position was assessed using an inclinometer (Table 3), a laser beam device (Table 4), and a smartphone goniometer (Table 5). All groups demonstrated measurable JPE values across all cervical movement directions (flexion, extension, right lateral bending, and left lateral bending), indicating the presence of cervical repositioning error. However, no statistically significant differences were observed among the four text neck severity groups for any movement direction, regardless of the measurement instrument used (*p* > 0.05 for all comparisons). Median comparisons are illustrated in Figure 14, Figure 15 and Figure 16. Similarly, JPE measurements obtained in the sitting position showed measurable repositioning errors across all groups. No statistically significant differences were found among the four groups for any cervical movement direction when assessed using the inclinometer (Table 6), laser beam device (Table 7), or smartphone goniometer (Table 8) (*p* > 0.05 for all comparisons). Although slight variations in JPE values were observed, these differences were not statistically significant. Median comparisons are presented in Figure 17, Figure 18 and Figure 19. Spearman’s rank correlation analysis was conducted to examine the relationship between text neck severity and JPE values measured by the three instruments in both standing and sitting positions (Table 9, Table 10 and Table 11). The results demonstrated weak and predominantly non-significant correlations across all cervical movement directions and measurement methods. In the standing position, flexion measured using the inclinometer showed a weak positive correlation (r = 0.22, *p* = 0.04); however, the strength of this association was clinically negligible. All other correlations were weak (r ranging from −0.24 to 0.08) and not statistically significant (*p* > 0.05).

## 4. Discussion

The primary objective of this study was to determine whether cervical proprioception, as measured by joint position error (JPE), differs among university students with varying degrees of text neck, and to examine the relationship between text neck severity and JPE. Participants were categorized into four groups: normal posture (no text neck), mild, moderate, and severe based on observational assessments conducted in both sitting and standing positions. JPE was evaluated in both postures, acknowledging that biomechanical and neuromuscular demands differ between them. Sitting posture relies more heavily on cervical proprioceptive input to maintain head and trunk alignment, whereas postural control in standing is influenced to a greater extent by proprioceptive inputs from the lower extremities, particularly the ankle and hip joints. This rationale is consistent with findings reported by Kim et al. and Shaghayegh et al. [7,16]. Similarly, Mashabi et al. reported that text neck severity varies depending on posture (sitting versus standing) and academic discipline, but may not independently predict cervical joint position error. Their findings highlight the importance of assessing both postures to obtain a comprehensive understanding of cervical sensorimotor function [6]. The results of the present study demonstrated that JPE was present across all participant groups, including those with normal posture, in both sitting and standing conditions. However, no statistically significant differences were observed among the four text neck severity groups. Furthermore, correlation analyses revealed no significant association between text neck severity and JPE across all movement directions and measurement tools. The presence of proprioceptive errors in individuals with normal cervical posture may be explained by central rather than peripheral factors. Central fatigue can impair the processing and integration of afferent input, even in the absence of peripheral dysfunction. University students, particularly during periods of academic stress, may be especially susceptible to central fatigue, which can negatively affect attention, motor control, and sensorimotor integration. This interpretation is supported by Abd-Elfattah et al. [17], who demonstrated that fatigue adversely affects both cognitive and neuromuscular performance. In addition, reduced physical activity may contribute to diminished proprioceptive function. Most participants in this study reported sedentary lifestyles, which are associated with decreased neuromuscular efficiency and impaired sensorimotor control. Previous studies by Kamijo et al. and Tomporowski have shown that physical inactivity is linked to deficits in cognitive processing and motor performance, which may in turn affect proprioceptive accuracy [18,19]. Other contributing factors may include psychological stress, disrupted sleep, and visual fatigue, conditions commonly observed among university students, particularly during examination periods. These factors can negatively influence central nervous system function and sensorimotor coordination. Prolonged use of electronic devices, such as smartphones and laptops, may also lead to chronic visual strain, further interfering with proprioceptive feedback and motor control. These findings are consistent with previous research by Williamson and Feyer, Bagesteiro et al., and Alshahrani et al. [20,21,22]. Collectively, these findings suggest that proprioceptive deficits observed in individuals with normal posture are likely influenced by multifactorial central and behavioral factors, rather than being solely dependent on structural or postural abnormalities. In participants with text neck, muscular fatigue may represent a key contributing factor to proprioceptive impairment. Fatigue in the cervical musculature can alter muscle spindle sensitivity and afferent discharge, thereby impairing central integration of proprioceptive signals. The accumulation of metabolic byproducts may further disrupt neuromuscular function. These mechanisms have been well documented in previous studies by Caron, Vuillerme et al., Moustafa et al., and Sajjadi et al. [23,24,25,26]. However, the findings of the present study differ from those of Reddy et al., who reported significant differences in JPE in a clinical population. This discrepancy may be attributed to differences in participant characteristics. Reddy et al. included individuals aged 20–50 years and focused on dentists, a population exposed to sustained cervical strain, whereas the present study included only healthy female students aged 18–25 years and excluded dental students [27]. Methodological differences may also explain the variation in findings. For example, Lee et al. used repeated laser-based JPE measurements, which may have introduced learning or correction effects. In contrast, the present study employed a single-trial protocol following a brief period of position memorization, which may better reflect natural proprioceptive performance in real-world conditions [28]. The absence of a significant correlation between text neck severity and JPE suggests that forward head posture alone may not be sufficient to produce measurable proprioceptive deficits in asymptomatic young adults. This may be explained by preserved neuromuscular adaptability and compensatory mechanisms that maintain sensorimotor control despite postural deviations. This finding is consistent with evidence indicating that proprioceptive impairments are more pronounced in individuals with neck symptoms rather than in those with postural deviations alone. Alternatively, the lack of association may reflect limitations in measurement sensitivity. Although the inclinometer, smartphone goniometer, and laser beam device are considered valid and reliable tools, they may not detect subtle proprioceptive impairments in asymptomatic individuals. This interpretation is supported by De Zoete et al. [29], who highlighted variability in the measurement properties of JPE assessments. Additionally, the use of a single trial per movement direction may have limited the ability to capture intra-individual variability in repositioning accuracy. Therefore, both physiological resilience in the studied population and limitations in measurement sensitivity should be considered when interpreting these findings.

### Study Limitation

This study has several limitations that should be acknowledged. Firstly, the cross-sectional design prevents establishing causality between text neck severity and cervical joint position error (JPE). Secondly, although the overall sample size was adequate, the relatively small number of participants within each subgroup may have limited the statistical power to detect subtle between-group differences. Thirdly, the sample was limited to female university students from a single institution, which may reduce the generalizability of the findings to other populations, including males, different age groups, and occupational settings. Additionally, the assessment of text neck severity relied on visual postural examination and angle-based classification, which, although standardized, may still be influenced by observer subjectivity. Moreover, while the tools used to assess JPE (laser beam, inclinometer, and smartphone goniometer) are considered reliable, variations in measurement techniques and participant compliance could have affected the accuracy of the results. Based on these limitations, future research is recommended to adopt longitudinal designs to explore causal relationships and monitor changes in proprioception over time. Expanding the sample to include a more diverse population would enhance external validity. It is also recommended to standardize measurement protocols across evaluators and consider combining objective assessments with imaging or neuromuscular evaluations to better understand underlying mechanisms. Finally, investigating the effects of proprioception-targeted interventions in individuals with text neck may provide important insights for clinical application.

## 5. Conclusions

In conclusion, this study did not demonstrate a statistically significant association between text neck severity and cervical joint position error (JPE) among young asymptomatic female university students. Although measurable repositioning errors were observed across all groups, JPE values did not differ significantly according to posture severity in either standing or sitting positions. Correlation analysis revealed predominantly weak and non-significant relationships between text neck severity and proprioceptive performance, suggesting limited evidence of sensorimotor impairment associated with postural classification in this population. The three assessment tools, the laser beam device, the inclinometer, and the smartphone goniometer, produced consistent findings, supporting their utility in measuring cervical repositioning error. However, the absence of strong associations indicates that text neck severity alone may not be a sufficient predictor of proprioceptive deficits in asymptomatic young females. These findings are specific to the studied population of young asymptomatic female university students. Further longitudinal research with larger and more diverse samples is recommended to explore potential causal mechanisms and to determine whether prolonged exposure to forward head posture contributes to sensorimotor alterations over time.

## Figures and Tables

**Table 1:** The median values of the demographic data for the four groups from the standing positions.

Variables	Normal (n = 8)		Mild Text Neck (n = 37)		Moderate Text Neck (n = 14)		Severe Text Neck (n = 9)		K TEST	*p* Value
	Median	IQR	Median	IQR	Mean	IQR	Median	IQR		
Age (yo)	21.56	1.01	21.24	1.44	20.64	1.28	21.44	1.04	4.46	0.22
Weight (kg)	51.04	12.17	52.78	9.55	45.92	9.78	52.64	13.04	6.09	0.11
Height (cm)	156.56	5.25	156.86	6.45	155.89	8.044	157.78	10.89	0.448	0.93
BMI (kg/h^2^)	20.85	4.93	21.41	3.32	19.15	5.695	21.65	7.39	8.03	0.05

IQR: interquartile ranges, yo: years old, kg: kilogram, cm: centimeter, h: height, n: number of students, K: Kruskal–Wallis test. Statistical significance was set at *p* < 0.05.

**Table 2:** The median values of demographic data for the four groups from the sitting position.

Variables	Normal (n = 11)		Mild Text Neck (n = 33)		Moderate Text Neck (n = 14)		Severe Text Neck (n = 10)		K Test	*p* Value
	Median Values	IQR	Median Values	IQR	Median Values	IQR	Median Values	IQR		
Age (yo)	21.67	0.78	21.06	1.39	21.29	1.64	20.93	1.11	4.01	0.26
Weight (kg)	49.43	11.31	51.59	9.67	55.49	11.82	45.63	9.09	6.04	0.11
Height (cm)	157	4.84	156.19	6.64	158	7.94	156.52	10.59	2.46	0.48
BMI (kg/h^2^)	20.02	4.36	21.13	3.49	22.44	5.89	19.11	6.49	7.34	0.06

(IQR: interquartile ranges, yo: years old, kg: kilogram, cm: centimeter, h: height, n: number of students, K: Kruskal–Wallis test. Statistical significance was set at *p* < 0.05).

**Table 3:** The inclinometer median values JPE for all four groups from the standing position.

Variables	Normal		Mild Text Neck		Moderate Text Neck		Severe Text Neck		K Test	*p* Value
	Median	IQR	Median	IQR	Median	IQR	Median	IQR		
Flexion	3.61	1.92	3.82	1.91	3.64	2.33	3.52	2.51	0.13	0.99
Extension	3.82	1.91	3.98	1.82	4.13	1.53	3.71	1.52	0.46	0.93
Right side bending	3.74	1.73	3.37	1.53	3.84	1.84	2.01	1.73	3.66	0.30
Left side bending	3.85	1.85	2.65	1.72	3.85	1.92	3.12	1.94	3.75	0.28

IQR: interquartile ranges, K: Kruskal–Wallis test. Statistical significance was set at *p* < 0.05.

**Table 4:** The laser beam median values JPE for all four groups from the standing position.

Variables	Normal		Mild Text Neck		Moderate Text Neck		Severe Text Neck		K Test	*p* Value
	Median Values	IQR	Median Values	IQR	Median Values	IQR	Median Values	IQR		
Flexion	11.46	6.17	9.51	5.57	7.70	3.45	7.65	6.34	2.33	0.51
Extension	7.74	4.53	7.65	4.32	6.73	4.03	8.91	5.33	0.495	0.92
Right side bending	7.49	4.72	6.50	3.89	8.82	4.11	6.14	4.44	4.13	0.25
Left side bending	6.69	4.87	6.29	4.52	6.59	2.26	6.68	3.67	1.03	0.79

IQR: interquartile ranges, K: Kruskal–Wallis test. Statistical significance was set at *p* < 0.05.

**Table 5:** The smartphone goniometer median values JPE for all four groups from the standing position.

Variables	Normal		Mild Text Neck		Moderate Text Neck		Severe Text Neck		K Test	*p* Value
	Median Values	IQR	Median Values	IQR	Median Values	IQR	Median Values	IQR		
Flexion	3.11	1.24	2.63	1.91	1.71	0.81	2.65	2.51	3.49	0.32
Extension	5.32	2.33	2.73	1.92	3.22	1.21	4.54	2.72	4.12	0.23
Right side bending	3.23	1.92	3.54	1.84	2.42	1.72	2.46	2.22	5.61	0.12
Left side bending	3.11	1.71	2.91	1.82	3.11	1.82	2.66	1.24	0.21	0.98

(IQR: interquartile ranges, K: Kruskal–Wallis test. Statistical significance was set at *p* < 0.05).

**Table 6:** The inclinometer median values JPE for all four groups from the sitting position.

Variables	Normal		Mild Text Neck		Moderate Text Neck		Severe Text Neck		K Test	*p* Value
	Median Values	IQR	Median Values	IQR	Median Values	IQR	Median Values	IQR		
Flexion	3.31	1.94	3.75	2.12	3.72	1.75	3.83	2.13	0.25	0.97
Extension	3.72	1.73	3.84	1.74	2.85	1.64	3.53	1.93	2.92	0.41
Right side bending	4.13	1.22	3.23	1.73	2.96	1.93	2.63	1.62	3.28	0.35
Left side bending	1.84	0.91	2.62	1.73	2.47	2.31	2.12	1.52	1.28	0.73

IQR: interquartile ranges, K: Kruskal–Wallis test. Statistical significance was set at *p* < 0.05.

**Table 7:** The laser beam median values JPE for all four groups from the sitting position.

Variables	Normal		Mild Text Neck		Moderate Text Neck		Severe Text Neck		K Test	*p* Value
	Median Values	IQR	Median Values	IQR	Median Values	IQR	Median Values	IQR		
Flexion	11.13	5.08	10.56	6.25	12.69	7.15	9.44	5.54	1.19	0.75
Extension	8.68	5.77	10.29	6.64	12.87	7.91	7.99	4.25	2.92	0.41
Right side bending	10.24	4.91	9.57	5.54	11.95	6.29	8.45	2.79	0.85	0.84
Left side bending	7.41	5.31	9.09	5.94	7.64	3.76	7.53	1.71	2.07	0.56

IQR: interquartile ranges, K: Kruskal–Wallis test. Statistical significance was set at *p* < 0.05.

**Table 8:** The smartphone goniometer median values JPE for all four groups from the sitting position.

Variables	Normal		Mild Text Neck		Moderate Text Neck		Severe Text Neck		K Test	*p* Value
	Median Values	IQR	Median Values	IQR	Median Values	IQR	Median Values	IQR		
Flexion	3.13	1.35	2.73	1.56	3.21	1.93	3.44	1.91	1.43	0.69
Extension	3.43	2.46	2.94	1.85	2.52	1.22	2.73	1.62	0.44	0.93
Right side bending	3.34	2.17	3.78	2.14	3.22	1.21	2.22	1.43	2.42	0.49
Left side bending	2.42	1.58	2.93	1.53	2.33	1.56	3.24	1.62	3.20	0.36

IQR: interquartile ranges, K: Kruskal–Wallis test. Statistical significance was set at *p* < 0.05.

**Table 9:** The Spearman correlation between text neck severity and inclinometer JPE for neck motions.

Variables	Standing Position r	*p*-Value	Sitting Position r	*p*-Value
Flexion	0.22	0.04	0.06	0.64
Extension	0.08	0.47	−0.14	0.30
Right side bending	0.05	0.65	−0.24	0.08
Left side bending	0.02	0.85	−0.05	0.71

r: Spearman’s rank correlation coefficient. Statistical significance was set at *p* < 0.05.

**Table 10:** The Spearman correlation between text neck severity and smartphone JPE for neck motions.

Variables	Standing Position r	*p*-Value	Sitting Position r	*p*-Value
Flexion	−0.16	0.15	0.07	0.62
Extension	−0.02	0.83	−0.07	0.57
Right side bending	−0.19	0.08	−0.07	0.55
Left side bending	−0.18	0.09	0.03	0.82

r: Spearman’s rank correlation coefficient. Statistical significance was set at *p* < 0.05.

**Table 11:** The Spearman correlation between text neck severity and laser beam JPE for neck motions.

Variables	Standing Position r	*p*-Value	Sitting Position r	*p*-Value
Flexion	0.01	0.99	−0.01	0.96
Extension	−0.14	0.19	0.08	0.45
Right side bending	−0.05	0.62	0.03	0.80
Left side bending	0.08	0.49	−0.11	0.28

r: Spearman’s rank correlation coefficient. Statistical significance was set at *p* < 0.05.

## Data Availability

The data presented in this study are available upon request from the corresponding author. The data are not publicly available due to privacy and ethical restrictions related to participant confidentiality.
